# Peculiarities of the psychological state of patients with chronic non-infectious liver diseases

**DOI:** 10.1192/j.eurpsy.2021.662

**Published:** 2021-08-13

**Authors:** I. Koval, O. Khaustova, K. Skurat

**Affiliations:** Medical Psychology, Psychosomatic Medicine & Psychotherapy, Bogomolets National Medical University, Kiyv, Ukraine

**Keywords:** chronic non-infectious liver disease, Depression, Anxiety, social maladaptation

## Abstract

**Introduction:**

The prevalence of chronic liver diseases (CLD) is over 30 million people worldwide, they are associated with significant health care costs, loss of productivity of patients, and has a significant impact on the quality of life associated with health.

**Objectives:**

The research objective was to determine current views on the psychological state of patients with CLD.

**Methods:**

A qualitative and quantitative analysis of the content of scientific Ukrainian and English literature published from 2014 to 2020, which sets out different views on the psychological state of patients with chronic liver diseases using the PubMed and Google Scholar databases. Only concept analysis, meta-analysis, and systematic reviews published in English, presented in the scientific literature were included.

**Results:**

The information on the peculiarities of the psychological state of patients with CLD was generalized. Based on the research, we can conclude that this group of patients is characterized by low mood, chronic fatigue, low level of social adaptation, increased anxiety, and reduced efficiency.

**Conclusions:**

Studies by different scientists from different countries agree that patients with chronic liver disease are characterized by the above symptoms. Some emphasize psychoneurophysiology and associate these symptoms with chronic inflammation, which occurs as liver damage progresses. Other researchers suggest that it is due to the quality of life of these patients and the severity of the disease. However, the scientific community has yet to find out what exactly caused this.

